# Tuning Pt-CeO_2_ interactions by high-temperature vapor-phase synthesis for improved reducibility of lattice oxygen

**DOI:** 10.1038/s41467-019-09308-5

**Published:** 2019-03-25

**Authors:** Xavier Isidro Pereira-Hernández, Andrew DeLaRiva, Valery Muravev, Deepak Kunwar, Haifeng Xiong, Berlin Sudduth, Mark Engelhard, Libor Kovarik, Emiel J. M. Hensen, Yong Wang, Abhaya K. Datye

**Affiliations:** 1 Voiland School of Chemical Engineering and Bioengineering, Washington State University, Pullman, Washington 99164 USA; 2 Department of Chemical and Biological Engineering and Center for Micro-Engineered Materials, University of New Mexico, Albuquerque, New Mexico 87131 USA; 3 Laboratory of Inorganic Materials and Catalysis, Department of Chemical Engineering and Chemistry, Eindhoven University of Technology, P.O. Box 513, 5600 MB Eindhoven, The Netherlands; 4 Environmental Molecular Sciences Laboratory, Pacific Northwest National Laboratory, Richland, Washington 99354 USA; 5 Institute for Integrated Catalysis, Pacific Northwest National Laboratory, Richland, Washington 99354 USA

## Abstract

In this work, we compare the CO oxidation performance of Pt single atom catalysts (SACs) prepared via two methods: (1) conventional wet chemical synthesis (strong electrostatic adsorption–SEA) with calcination at 350 °C in air; and (2) high temperature vapor phase synthesis (atom trapping–AT) with calcination in air at 800 °C leading to ionic Pt being trapped on the CeO_2_ in a thermally stable form. As-synthesized, both SACs are inactive for low temperature (<150 °C) CO oxidation. After treatment in CO at 275 °C, both catalysts show enhanced reactivity. Despite similar Pt metal particle size, the AT catalyst is significantly more active, with onset of CO oxidation near room temperature. A combination of near-ambient pressure X-ray photoelectron spectroscopy (NAP-XPS) and CO temperature-programmed reduction (CO-TPR) shows that the high reactivity at low temperatures can be related to the improved reducibility of lattice oxygen on the CeO_2_ support.

## Abstract (web summary)

While single-atom catalysts (SACs) have attracted a lot of interest, the nature of the active sites in SACs remains elusive. Here the authors elucidate that depositing single atoms via high temperature synthesis leads to improved reducibility of lattice oxygen on CeO2 yielding low temperature reactivity of Pt catalysts in CO oxidation.

## Introduction

The U.S. Department of Energy (DOE) road map has set the goal for automotive treatment technologies to achieve 90% conversion of criteria pollutants at a temperature (T_90_) of 150 °C or lower^1^. In addition to high reactivity at low operating temperatures, thermal durability is essential to survive harsh conditions encountered in automotive exhaust. One strategy to achieve high reactivity is to use single atom catalysts (SACs) which also provide efficient utilization of scarce platinum group metals (PGMs). Current synthesis methods such as strong electrostatic adsorption (SEA), ion exchange, co-precipitation, impregnation or deposition-precipitation use low metal loading (~0.2 wt.%) to generate single-atom catalysts (SACs)^2–5^. Generally, operating temperatures with SACs are limited (<300 °C) to prevent agglomeration of single atoms into nanoparticles^3,6,7^. SACs with excellent thermal stability are of relevance to automotive emission treatment technologies in which PGMs are used. The International Organization of Motor Vehicle Manufacturers (OICA) reports that the world vehicle production in 2016 was over 95 million^8^. To increase efficiency and reduce costs it would be desirable to keep the PGMs highly dispersed, even when the catalyst is subjected to temperatures as high as 800 °C during accelerated aging protocols^9^.

Ceria is a commonly used component in automotive exhaust catalysts. We recently showed that ceria can provide sites for trapping Pt single atoms which are thermally stable at 800 °C in air^10^. Hence, this method of synthesis meets the requirements of the DOE protocol for accelerated aging of diesel oxidation catalysts (DOCs). However, the atomically dispersed, ionic form of Pt is not active for low-temperature CO oxidation^10^. This was already recognized in 1992 by Nunan et al. who pointed out that catalyst activity declined after oxidation, and reduction of the catalyst was necessary to achieve high reactivity^11^. Gänzler et al.^12^ used extended X-ray absorption fine structure (EXAFS) and environmental transmission electron microscopy (ETEM) to demonstrate that formation of Pt nanoparticles was necessary to achieve high reactivity. In a later study, Gänzler et al.^13^ concluded that 1.4 nm Pt particles represent the optimal size. On the other hand, Gatla et al.^14^ concluded that to achieve room temperature CO oxidation activity, the Pt particles should be very small. The characteristic of these small particles was their tendency to spread when exposed to the electron beam. In their recent work, Gatla et al.^15^ report that the particle size of Pt in the most active catalyst (reduced at 300 °C in H_2_) is too small to detect via TEM. The XPS data shows Pt^2+^ species, so they argue that the oxidized Pt may be responsible for the high reactivity. Since XPS was performed on air-exposed samples, it is possible that Pt got oxidized during transfer in air. It is clear that there is a need for in-situ XPS studies under conditions of low-temperature CO oxidation, which will help elucidate the nature of the active sites.

With regard to Pt particle size, it is well known that the vibrational frequency of adsorbed CO decreases with decreasing particle size of Pt, and the strength of CO binding increases^16,17^. Hence, very small particles may not be desired for this reaction since they will be subject to strong CO binding leading to CO poisoning. Indeed, Gänzler et al.^13^ suggest that very small Pt particles (and single atoms) may not be effective. However, other work in the literature suggests that even isolated single atoms of Pt^3,18^ or corner atoms on Pt nanoparticles^19^ should be active for CO oxidation. It is clear, then, particle size may not be the sole factor for achieving high CO oxidation activity. The other important factor is the nature of the support, its ability to provide oxygen species to the active sites. The unique ability of ceria to change oxidation state from Ce^4+^ to Ce^3+^ and its role on CO oxidation kinetics were originally demonstrated by Bunluesin et al.^20–22^. In recent work, Kopelent et al.^23^ pointed out that ceria reduction is a kinetically relevant step during CO oxidation. Previous studies have focused on the size of ceria particles, or their exposed surface facets to improve CO oxidation activity^24–26^. Since the reducibility of ceria is influenced by the nature of metal-support interaction, in this work two methods of catalyst preparation were studied, leading to different extent of interaction between Pt and CeO_2_.

A conventional catalyst preparation involving adsorption of the precursor on the support (SEA)^4^ was used with a moderate calcination temperature (350 °C in air). With the SEA catalyst preparation, the Pt precursor is initially bound to the surface through ligands that are removed during calcination^27^. In contrast, the method of AT involves calcination of the adsorbed precursor at 800 °C in air which causes the Pt to form covalent bonds with the surface oxygen atoms^28,29^. The high-temperature synthesis can also cause restructuring of the ceria support as ceria becomes mobile and loses surface area at the high temperatures^10^. In this work we also varied the catalyst precursor (chloroplatinic acid–CPA and tetraammineplatinum nitrate–TAPN) to understand its influence on catalytic activity. The nature of the catalysts was studied by High-angle annular dark-field scanning transmission electron microscopy (HAADF-STEM), diffuse reflectance infrared Fourier transform spectroscopy (DRIFTS), CO-TPR, NAP-XPS and Raman spectroscopy. We used CO-TPR and NAP-XPS to study the ease of reducibility of ceria and its ability to provide oxygen to the active sites.

The results presented here show that the catalyst prepared via AT synthesis achieves the highest reactivity after reduction in CO at 275 °C. Spectroscopic and microscopic characterization suggests that the AT synthesis leads to a stronger interaction between Pt and ceria, which results in the activation of support oxygen at lower temperatures. The AT catalyst retains a portion of the initial ionic Pt species after CO reduction at 275 °C, but most of it is lost when the catalyst is reduced at 450 °C. Consequently, the reactivity drops after this harsh reduction. In-situ DRIFTS shows that during CO oxidation, the adsorbed CO on the Pt nanoparticles in the 275 °C-activated AT catalyst is lost readily when CO flow is stopped, and helium is flowed. The results help explain why the onset of CO oxidation occurs near room temperature, a temperature at which metallic Pt is poisoned by CO. We demonstrate that preparation of the catalyst via high-temperature vapor-phase synthesis restructures the ceria, enhancing its reducibility and making it possible for the catalyst to perform well under redox conditions present in the exhaust of internal combustion engines.

## Results

### CO oxidation activity of the AT and SEA catalysts

**Fig. 1:**
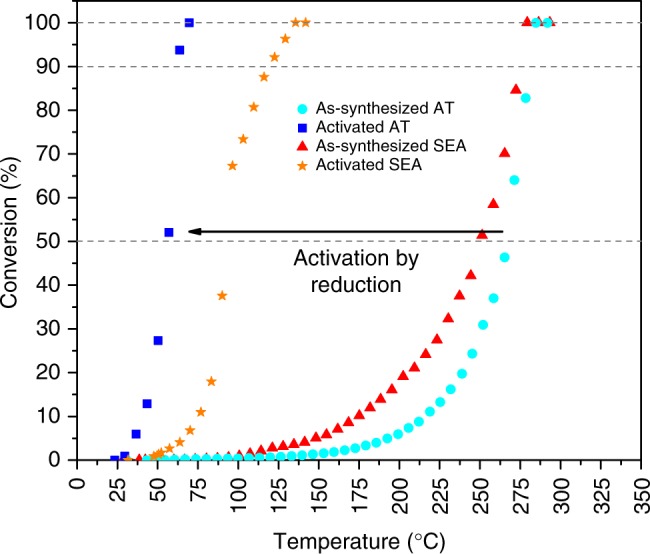
CO oxidation light-off curves for 1wt.%Pt/CeO_2_ catalysts. The activity of the catalysts synthesized by AT and SEA methods was measured before and after activation. TAPN was used as precursor

Figure 1 shows CO oxidation light-off curves for the catalysts synthesized by AT and SEA before activation (called as-synthesized) and the corresponding catalysts that were further reduced at 275 °C in CO (called activated). The activity of the as-synthesized catalysts is very low, both showing a T_90_ of ~280 °C which has been observed previously on isolated ionic Pt species supported on ceria^10,12^. The activated AT catalyst shows significantly higher reactivity and reaches 90% conversion at 64 °C, while the activated SEA catalyst achieves 90% conversion at 120 °C. The same trend was observed for the AT and SEA catalysts synthesized using CPA as precursor (Supplementary Fig. 1). Furthermore, the activated AT catalyst shows no deactivation after numerous CO oxidation runs (Supplementary Fig. 2). It is also important to consider that this catalyst was synthesized by heating in air at 800 °C which satisfies the requirements for accelerated aging set by the U.S. DRIVE partnership for DOCs^9^. Moreover, it is worth noting that the support alone does not contribute to the reactivity as shown in Supplementary Fig. 3.

Supplementary Table 1 shows the turnover frequency (TOF) (calculated with respect to the total amount of Pt) at 80 °C and activation energy for the as-synthesized and activated AT and SEA catalysts. Both activated catalysts have higher TOF than the as-synthesized catalysts. This is more noticeable in the AT catalyst in which the TOF of the activated catalyst (at 80 °C) is ~20 times while the SEA is only ~6 times higher than the corresponding as-synthesized catalysts. Moreover, the catalyst prepared by AT exhibits a decrease in activation energy from 53.5 to 30.1 kJ/mol after activation. This suggests that the nature of the active site and the reaction mechanism on the activated AT catalyst could be different. The SEA catalyst also shows a decrease in the activation energy after activation, but to a lesser extent.

### Atomic-scale images of catalysts after activation

**Fig. 2:**
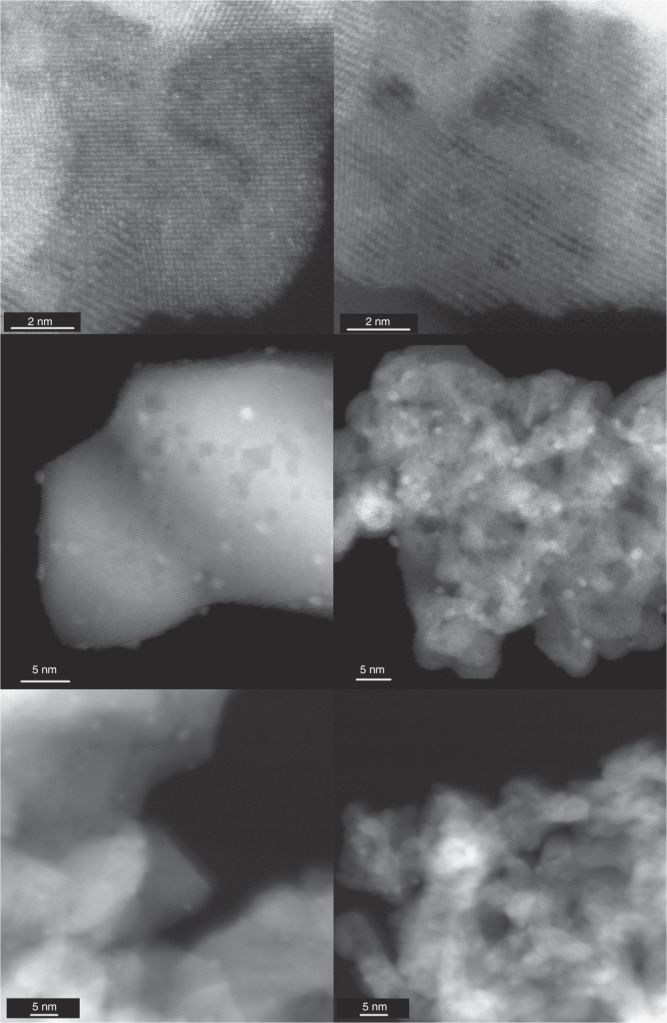
HAADF-STEM images of the 1wt.% Pt/CeO_2_ catalysts: **a** as-synthesized AT (CPA), **b** as-synthesized SEA (CPA), **c** activated AT (CPA), **d** activated SEA (CPA), **e** activated AT (TAPN), **f** activated SEA (TAPN)

Figure 2 shows HAADF-STEM images of the as-synthesized (CPA) and activated (CPA and TAPN) catalysts. In the as-synthesized state, the AT and SEA catalysts show only Pt single atoms. After activation, Pt transforms into nanoparticles, exhibiting very similar particle size for AT and SEA catalysts. Supplementary Figs. 4 and 5 show the particle size distribution (PSD) for the activated TAPN and CPA catalysts. Results indicate that in the TAPN catalysts, Pt nanoparticles have a mean particle size and standard deviation of 1.68 ± 0.3 and 1.58 ± 0.33 nm, for the AT and SEA catalysts, respectively, while in the case of the CPA catalysts, these values are 1.05 ± 0.2 nm and 1.72 ± 0.36 nm, respectively. Therefore, differences in reactivity between AT and SEA catalysts cannot be attributed to differences in the Pt particle size, which are comparable, and all under 2 nm, the size at which Cargnello et al.^19^ observed the highest reactivity in their study of particle size effects. Negative effects of chloride on the activity of ceria-supported transition metal catalysts have been reported previously^30,31^. Hence, to avoid incorrect conclusions due to the effects of Cl in the catalysts, the main focus for the rest of the manuscript will be on the TAPN catalysts.

### Probing the Pt-CO interaction with infrared spectroscopy

**Fig. 3:**
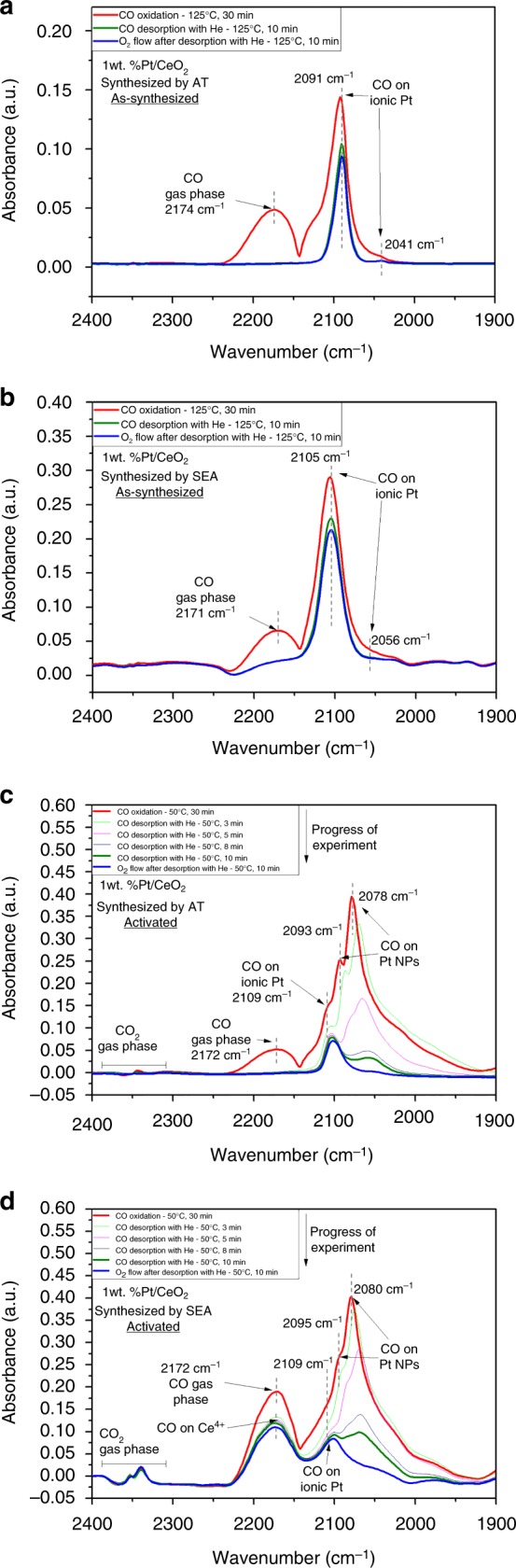
CO oxidation reaction monitored by DRIFTS on the 1wt% Pt/CeO_2_ TAPN catalysts: **a** as-synthesized AT (125 °C), **b** as-synthesized SEA (at 125 °C), **c** activated AT (50 °C), **d** activated SEA (50 °C)

CO oxidation was performed, and the adsorbed species were monitored via DRIFTS to probe the surface of the as-synthesized and activated catalysts. The as-synthesized AT catalyst shows a well-defined peak at 2091 cm^−1^ with a small shoulder at 2041 cm^−1^ (Fig. 3a). The as-synthesized SEA catalyst is similar, with a prominent peak at 2105 cm^−1^ and a shoulder at 2056 cm^−1^ (Fig. 3b). Based on previous work^10^, the peaks at high wavenumber (2091 and 2105 cm^−1^) can be assigned to CO adsorbed on ionic Pt species corresponding to Pt single atoms supported on CeO_2_, but the difference in peak position between catalysts suggests there is a different interaction between the metal and the support. There is little change in the peak intensity after CO was stopped and helium or oxygen were flowed, indicating that CO is strongly bound to the ionic Pt. This results in low activity for both catalysts in the as-synthesized state (CO poisoning)^32^, which matches the activity results observed in Fig. 1. This strong bond between CO and ionic Pt single atoms has been observed previously^33^. Even though the peaks at low wavenumber (2046 cm^−1^ and 2051 cm^−1^) fall in the range for CO on Pt nanoparticles, the CO adsorbs strongly to Pt on these sites and is not removed even after helium or oxygen were flowed. On the other hand, the fact that CO adsorbed on metallic Pt nanoparticles reacts readily with oxygen suggests the peaks at low wavenumber can also be assigned to oxidized Pt sites^17,33^.

Figure 3c and d show spectra for CO oxidation at 50 °C for the activated AT and SEA catalysts, respectively. At this temperature, the AT catalyst shows ~20% conversion of CO, while the SEA catalyst shows <3% CO conversion. The activated AT catalyst shows two prominent bands observed at 2093 cm^−1^ and 2078 cm^−1^ due to CO adsorption on metallic Pt nanoparticles^33–47^. These peaks disappear readily as soon as the CO flow is stopped, and helium is flowed, indicating that CO is weakly bound to Pt nanoparticles. A smaller peak is observed at 2109 cm^−1^ and is assigned to CO adsorbed on ionic Pt. This site binds CO very strongly since the peak does not disappear after helium or oxygen is flowed. Nevertheless, the nature of these ionic Pt species is likely to be different from the ionic Pt single atoms observed in the as-synthesized state. This is supported by the blueshift in the wavenumber compared to the peak in the as-synthesized state for ionic Pt single atoms and an observable redshift during desorption. Similar results have been obtained previously^18^, and the authors attributed this peak to Pt oxide clusters. Spectra during the activation treatment at 275 °C with CO (Supplementary Fig. 6) and spectra for CO adsorption at 125 °C before CO oxidation (Supplementary Fig. 7) do not show the same peak as observed in Fig. 3c. This suggests that the peak at 2109 cm^−1^ is formed during CO oxidation due to exposure to oxygen, supporting its assignment to Pt oxide clusters. The activated SEA catalyst shows similar behavior to the activated AT catalyst. Peaks for CO on Pt nanoparticles are observed at 2095 cm^−1^ and 2080 cm^−1^, while a peak for CO on ionic Pt is also observed at 2109 cm^−1^. The main difference between the two catalysts is that the intensity of the peaks for CO on Pt nanoparticles decreases more slowly for the SEA catalyst when the gas is switched to helium (Supplementary Table 2). CO adsorption on Ce^4+^ seen at 2172 cm^−1^^34^ is present in the activated SEA catalyst after helium or oxygen is flowed at 50 °C (Fig. 3d). This agrees with the activity differences since at 50 °C, the activated AT catalyst is able to provide oxygen at low temperatures, compared to the SEA catalyst. When the same experiment was performed at 125 °C (Supplementary Fig. 8), there was no significant difference in the disappearance of CO between the two catalysts, since both catalysts provide very high conversion of CO at this temperature. Therefore, DRIFTS results show that the increase in the reactivity after CO reduction is related to the formation of Pt nanoparticles and the ease with which adsorbed CO can be reacted away. These results are consistent with previous reports that CO on metallic Pt reacts readily with oxygen, unlike the CO on ionic Pt which does not react unless heated to higher temperatures^3,10,19,33^. Since the Pt particle size, as seen in Fig. 2, is similar on both catalysts, this difference in reactivity between the AT and SEA catalyst must be related to the catalyst support.

### Surface oxygen reactivity monitored by reduction with CO

Previous studies on ceria-supported Pt catalysts have observed that CO oxidation follows a Mars-Van Krevelen (MvK) reaction mechanism with positive effect of surface oxygen activation on the oxidation of CO^23,35,48^. It has also been observed that the relevant step during CO oxidation in a MvK reaction mechanism is the reaction between CO adsorbed on Pt and oxygen from the lattice^42^. Furthermore, previous work found that the amount and type of Pt species deposited on the support can play a role during the activation of surface oxygen^49,50^. Considering that the AT and SEA catalysts are synthesized by exposing them to significantly different temperatures, it is reasonable to expect a different interaction between Pt and CeO_2_ for the two catalysts. Therefore, CO-TPR was used to determine if there is a difference between the two catalysts in the reaction between CO and oxygen from the catalyst.

**Fig. 4:**
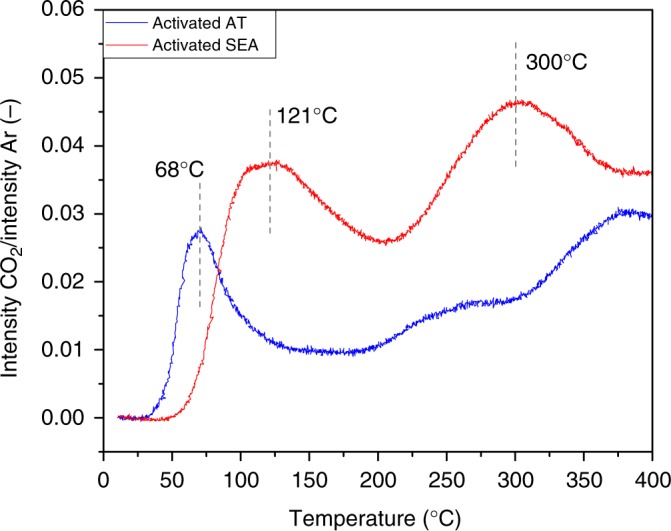
CO-TPR of the 1wt.%Pt/CeO_2_ catalysts. The reducibility of the catalysts synthesized by AT and SEA methods was monitored by observing the formation of CO_2_. Both catalysts were synthesized using TAPN as precursor and were activated prior to the experiment

The as-synthesized AT and SEA catalysts were exposed to CO to study the initial reduction of the catalyst, as reported in Supplementary Fig. 9. The ease of reducibility of the AT catalyst is evident in the as-synthesized catalyst. A separate aliquot of the catalyst was next activated via CO reduction at 275 °C, then exposed to an oxidation at 200 °C to remove any adsorbed species and to replenish the oxygen on the support. Figure 4 shows the formation of CO_2_ during CO-TPR for the activated catalysts. The AT catalyst shows CO_2_ formation at lower temperature than the SEA catalyst. Since both catalysts were exposed to an oxidative treatment prior to the CO-TPR, the CO_2_ must come from reactive oxygen species accessible during reaction. The results shown here demonstrate that the AT catalyst contains ceria sites that are reducible at low temperatures where the AT catalyst is active for CO oxidation. The SEA catalyst is only able to do this at higher temperatures. Previous work by Gatla et al.^14^ suggested that when there is a strong interaction between Pt and CeO_2_, the energy to remove the oxygen between Pt and Ce is reduced. Therefore, our results would suggest the interaction between Pt and CeO_2_ in the AT catalyst is stronger than in the SEA. This is not completely unexpected considering that the interaction between Pt and CeO_2_ has to be strong enough to keep Pt as single atoms during the calcination at 800 °C. These results are different from previous works in which the activation of oxygen is related to the previous formation of nanoparticles that help activate the reductant to further react with surface oxygen^12,13^. Here we show that high synthesis temperature is important to modify the ceria, allowing activation of surface oxygen at lower temperatures, which subsequently results in higher reactivity for CO oxidation at low temperature. Nevertheless, the high-temperature synthesis is beneficial only when Pt is already present on the support and the synthesis is made in an oxygen rich environment like air. Previous work by Bunluesin et al.^51^ showed that calcining only CeO_2_ at high-temperature (900 °C) and further depositing a metal, leads to low catalytic activity due a large increase in crystallite size. Therefore, it is not about exposing CeO_2_ to high temperatures to help activate oxygen at low temperature but rather promoting the interaction between Pt and CeO_2_ at high temperature. This interaction leads to volatile PtO_2_ being trapped on CeO_2_^10^, creating the active sites for oxygen activation at low temperature.

### Surface oxygen reactivity monitored by NAP-XPS

**Fig. 5:**
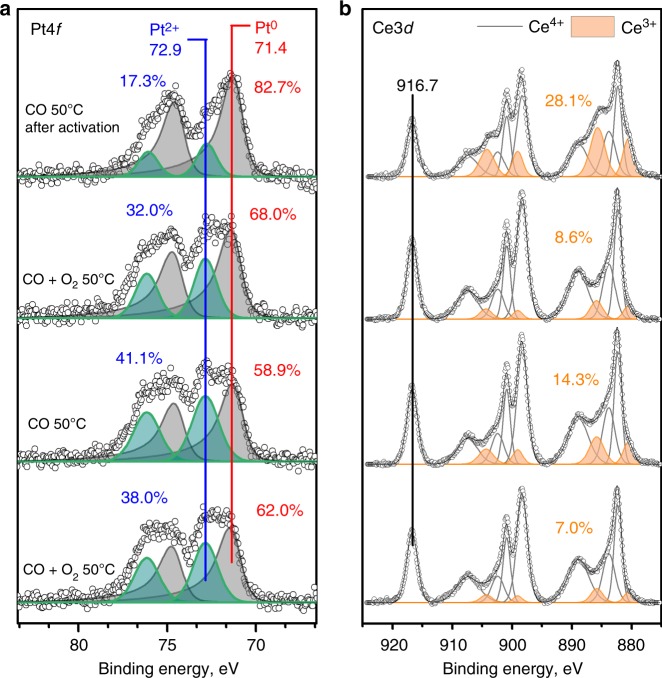
NAP-XPS results for the 1wt.%Pt/CeO_2_ TAPN catalyst synthesized by AT after activation in CO at 275 °C: **a** Pt4*f* region, **b** Ce3*d* region. The catalyst was sequentially exposed to different gas environments, starting at the top. The Pt/Ce ratio remained at ~0.030 throughout the experiment

To better understand the difference in activity between the AT and SEA catalysts, XPS was performed on the as-synthesized and activated states. Supplementary Fig. 10a, b show the Pt4*f* and Ce3*d* regions for the AT and SEA catalysts in the as-synthesized state. For both catalysts, only Pt^2+^ and Pt^4+^ species are observed^52,53^, confirming that the as-synthesized state only contains ionic Pt species. The percentage of Pt^2+^ species is very similar with 80.5% and 72.4% for the AT and SEA, respectively. On the other hand, the Ce^3+^ amount observed^54^ in the AT catalyst is almost double that of the SEA catalyst with 10.7% and 5.8%, respectively. Figure 5a, b show the Pt4*f* and Ce3*d* regions, respectively, for the activated AT catalyst after exposing it to different gas environments during NAP-XPS measurements (progress of experiment from top to bottom). Spectra after in-situ activation at 275 °C and then exposure to CO at 50 °C show that Pt^0^ is the primary species (% Pt^0^ ~ 83%). Continuously, exposing the catalyst to a mixture of CO and O_2_ at 50 °C leads to the reoxidation of some of the Pt species (% Pt^0^ = 68%). This is due to the small particle size of the Pt nanoparticles and supports the assignment of the 2109 cm^−1^ peak (Fig. 3c) to Pt oxide clusters. Further exposure to CO and CO + O_2_ environments at 50 °C do not change significantly the fraction of Pt^0^ species in the catalyst. On the other hand, the amount of Ce^3+^ species after activation with CO at 275 °C is ~28%. After exposing the catalyst to the reaction mixture at 50 °C, this fraction of Ce^3+^ decreases to 8.6%. Exposing the catalyst to CO at 50 °C increases the amount of Ce^3+^ to 14.3%, suggesting that even at 50 °C oxygen can be easily removed leaving behind vacancies and forming Ce^3+^ species. Further exposure to the reaction mixture at 50 °C reduces the fraction of Ce^3+^ species to 7%, suggesting these changes are reversible at 50 °C. The composition of the gas during the NAP-XPS experiments was monitored by mass spectrometry (MS) (Supplementary Fig. 11a) and it confirms the formation of CO_2_ when the catalyst is exposed to the reaction mixture at 50 °C.

**Fig. 6:**
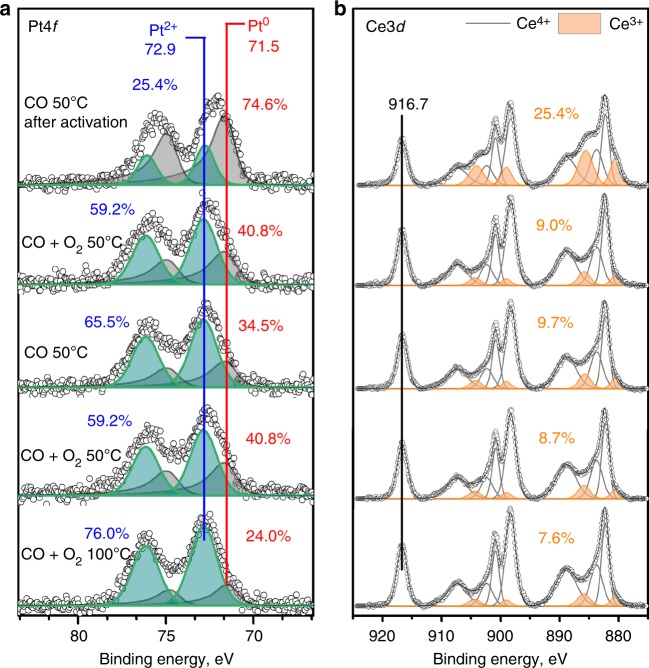
NAP-XPS results for the 1wt.%Pt/CeO_2_ TAPN catalyst synthesized by SEA after activation in CO at 275 °C: **a** Pt4*f* region, **b** Ce3*d* region. The catalyst was sequentially exposed to different gas environments, starting at the top. The Pt/Ce ratio remained at ~0.015 throughout the experiment

Figure 6a, b show the Pt4*f* and Ce3*d* regions, respectively, for the SEA catalyst activated with CO at 275 °C, after exposure to different gas environments during NAP-XPS (progress of experiment from top to bottom). The SEA catalyst also has Pt^0^ as the primary species (% Pt^0^ = 74.6%), however, compared to the AT catalyst, the SEA catalyst shows more reoxidation of the Pt species (% Pt^0^ = 40.8%) which remain stable after further exposure to CO and CO + O_2_ environments at 50 °C. The biggest difference between the AT and SEA catalysts is the reducibility of CeO_2_. The amount of Ce^3+^ after activation is similar to the AT catalyst (25.4%). Once the SEA catalyst is exposed to the reaction mixture at 50 °C, the Ce^3+^ species decrease to 9% and successive exposure to CO at 50 °C does not lead to a significant increase of Ce^3+^ (9.7%), suggesting that oxygen vacancies are not easily formed in the SEA catalyst under these low temperature reaction conditions, unlike on the AT catalyst. Further exposure to the reaction mixture leads to similar amount of Ce^3+^ species (8.7%). Since the SEA catalyst is less active than the AT, the temperature was increased to 100 °C and the catalyst was exposed to the reaction mixture. Results showed that the amount of Ce^3+^ decreases to 7.6%. Mass spectrometry (Supplementary Fig. 11b) confirms that a negligible amount of CO_2_ is formed at 50 °C while raising the temperature to 100 °C leads to a clearer formation of CO_2_, suggesting the catalyst is active at this temperature but not at 50 °C like the AT catalyst.

To probe the strength of the interaction between Pt and CeO_2_ for both catalysts, a harsh reduction treatment with CO at 450 °C for 8 h was performed (Supplementary Fig. 10c, d). Similar results are observed regarding the amount of Ce^3+^ species formed, however, the AT catalyst shows that a fraction of Pt^2+^ species is still present, while the SEA catalyst only shows Pt^0^ species. Therefore, the XPS results indicate that the interaction between Pt and CeO_2_ in the AT catalyst is stronger than in the SEA catalyst.

The active AT catalyst was further studied by in situ Raman spectroscopy (Supplementary Fig. 12). The catalyst in the as-synthesized state shows peaks at 555 cm^−1^, associated with Pt–O–Ce, and at 662 cm^−1^, attributed to Pt–O^55–57^. Activation of the catalyst leads to the reduction in the intensity of these peaks. This suggests that the low temperature peak observed during CO–TPR for the AT catalyst is associated with the reduction of the Pt-O-Ce bond. Previous theoretical studies on the reducibility of ceria-supported Pt catalysts showed that Pt^2+^ species had an effect on the reducibility of ceria. Pt^2+^ species can replace a Ce^4+^ cation and adopt a square-planar coordination, creating an oxygen vacancy and leaving three three-coordinate oxygen atoms that are easier to remove^58,59^. However, even though in our work both catalysts have ionic Pt species in the as-synthesized state, the bonds between Pt and CeO_2_ are different. Commonly, high-temperature treatments lead to the sintering and loss of surface area of the support along with the agglomeration of Pt, however, a strong interaction between Pt and ceria leads to mutual stabilization^10^. While Pt forms strongly bound ionic species on the support, these species also mitigate the sintering of ceria. Indeed, the presence of Pt causes the surface area of the AT catalyst to be ~30 m^2^/g higher after calcination at 800 °C (Supplementary Figs. 13, Table 3) than ceria that does not contain any Pt. It is worth noting that similar results were obtained when using the CPA precursor during synthesis. Supplementary Figs. 14 to 17 show the results for CO-TPR, DRIFTS, XPS and Raman spectroscopy, respectively. These results show that for the AT and SEA catalysts synthesized using the CPA precursor, a similar trend is observed.

## Discussion

**Fig. 7:**
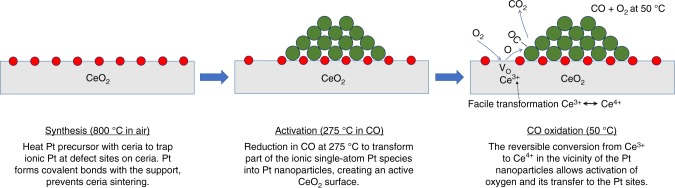
Synthesis of single-atom Pt/CeO_2_ with improved reducibility and its transformation into an active catalyst. The Pt/CeO_2_ single-atom catalyst is reduced and forms Pt nanoparticles. This active state of the catalyst readily provides oxygen to react with CO adsorbed on Pt nanoparticles, preventing CO poisoning. Red: ionic Pt, green: metallic Pt

Figure 7 shows a scheme describing the characteristics and transformations of the AT catalyst. The synthesis of the AT catalyst involves a high-temperature treatment (800 °C) which results in the stabilization of Pt single atoms on the surface of CeO_2_, as confirmed by STEM (Fig. 2a) and DRIFTS (Fig. 3a). Activation of the catalyst in CO at 275 °C leads to the partial transformation of Pt single atoms into Pt nanoparticles, also evidenced by STEM (Fig. 2c, e), DRIFTS (Fig. 3c) and NAP-XPS (Fig. 5). The NAP-XPS sees significant fraction of oxidized Pt species compared with DRIFTS. This is likely due to the presence of ionic Pt at the interface between metallic Pt nanoparticles and the support as well as the small size of the Pt particles and the escape depth of the photoelectrons. The ionic Pt at the interface will not be seen in DRIFTS, and since the extinction coefficient of the CO bands can vary significantly with Pt speciation, we find this NAP-XPS representation to reflect the expected state of the activated AT catalyst. The scheme in Fig. 7 also shows why the AT catalyst has high activity for low-temperature CO oxidation with a T_90_ of 64 °C (Fig. 1).The facile reaction of surface oxygen with CO adsorbed on the Pt nanoparticles is demonstrated via NAP-XPS (Fig. 5) at 50 °C where the fraction of Ce^3+^changes as oxygen is switched on and off. The creation and refilling of oxygen vacancies that can react with CO help explain the low temperature reactivity of the AT catalyst.

In contrast, the synthesis of the SEA catalyst involves a calcination at much lower temperature (350 °C) leading to weaker interaction with the ceria support. The as-synthesized state of the catalyst also shows Pt single atoms (Fig. 2b). Further activation of the SEA catalyst in CO at 275 °C leads to similar transformation of Pt single atoms into Pt nanoparticles (Figs. 2d, f, 3d and 6). Nevertheless, there is a clear difference in activity between the SEA and AT catalysts. NAP-XPS (Fig. 6) shows that unlike in the AT catalyst, CeO_2_ does not provide oxygen to react with CO at 50 °C, evidenced by the negligible change in the amount of Ce^3+^ species when exposed to CO and CO oxidation cycles. In other words, only when the Pt/CeO_2_ catalyst is synthesized by exposing it to air at high temperature (800 °C) and activated by CO reduction at 275 °C, does the oxygen in the support becomes more reactive at low temperatures. DRIFTS results (Fig. 3c, d) also show the difference between the AT and SEA catalysts after activation, in which after switching from CO oxidation to desorption with helium, the intensity of the bands ascribed to Pt nanoparticles decreases faster for the AT catalyst (Supplementary Table 2). After 10 min of desorption with helium, the intensity of the CO bands in the AT catalyst is only 9.5%, compared to 28% for the SEA catalyst. We attribute this difference to the facile reactivity of surface oxygen, confirmed by the lower reduction peak during CO-TPR for the AT catalyst (Fig. 4).

Additional benefits are observed during the synthesis at high temperature. The strong interaction between Pt and CeO_2_ required to stabilize Pt as single atoms also reduces the sintering of CeO_2_. This is observed by the larger BET surface area (Supplementary Fig. 13, Table 3) and smaller ceria crystallite size (X-Ray powder diffraction (XRD), Supplementary Fig. 18) of CeO_2_ in the Pt/CeO_2_ catalyst compared to pure CeO_2_. This stronger interaction between Pt and CeO_2_ in the AT catalyst is further confirmed by measuring the amount of ionic Pt single-atom species by XPS after a harsh reduction treatment in CO at 450 °C for 8 h (Supplementary Fig. 10c, d). Pt in the SEA catalyst is completely reduced while the AT catalyst still exhibits Pt^2+^ species. Even after such harsh reduction treatment Pt particle size did not increase significantly in the AT catalyst (1.87 ± 0.38 nm in Supplementary Fig. 19). However, a significant decrease in the activity was observed (Supplementary Fig. 20a), which can be explained by a loss of reactivity of surface oxygen at low temperature (Supplementary Fig. 20b). The loss of activity and reactivity of surface oxygen, along with a decrease in the amount of Pt single-atom species may suggest that ionic Pt single-atom species in the ceria, left behind after CO activation, are important for the activation of oxygen at low temperature.

The importance of small Pt clusters has been emphasized in the work of Cargnello et al.^19^ and Gänzler et al.^13^ who propose that the high activity for CO oxidation is related to the larger interfacial area which leads to more active sites for reaction between CO adsorbed on Pt and oxygen from the support. Gänzler et al.^13^ propose 1.4 nm as the optimal size, however in our work both the AT and SEA catalysts have particles in this same size range, but very different low temperature CO oxidation activity. We propose that particle size by itself is not an adequate descriptor for improved reactivity, especially when the particles are smaller than 2 nm. The reducibility of the support, which provides facile oxygen transfer to the metal particles is also important. The synthesis procedure presented here (AT) results in a stronger interaction between Pt and CeO_2_. This interaction allows stabilization of Pt nanoparticles under reaction conditions and more facile activation of oxygen from the support. As a result, a catalyst with enhanced activity for low-temperature CO oxidation is obtained.

The unique properties of the AT catalyst that lead to high reactivity for CO oxidation at low temperature open up the possibility to prepare similar catalysts for other oxidation reactions, to achieve high activity at lower temperatures. In summary, this work demonstrates the beneficial effect of high-temperature synthesis which allows restructuring of the catalyst support due to the strong interaction between the mobile metal precursor and the catalyst support, resulting in a catalyst with high thermal stability and high activity for low-temperature CO oxidation, making it suitable for industrial use.

## Methods

### Catalyst synthesis and activation

The 1wt.%Pt/CeO_2_ catalysts were synthesized by incipient wetness impregnation^10^. To prepare the 1wt.%Pt/CeO_2_ catalyst, cerium oxide powder support was synthesized via thermal decomposition of cerium (III) nitrate hexahydrate (Ce(NO_3_)_2_•6H_2_O, 99.999% purity, from Sigma Aldrich) at 350 °C for 2 h. The TAPN catalysts were synthesized using tetraammineplatinum nitrate (Sigma Aldrich 99.999% purity). After impregnation, the catalysts were directly calcined at 350 °C for 1 h and 800 °C 10 h, respectively, in flowing air. These two catalysts calcined at 350 and 800 °C are called ‘as-synthesized’ SEA and AT catalysts, respectively. To achieve a high-activity state, the catalysts are further reduced at 275 °C for 1 h using 6 ml/min CO and 70.5 ml/min of He. These catalysts are called ‘activated’ SEA and AT catalysts, respectively. CPA catalysts were synthesized using chloroplatinic acid solution 8 wt.% in H_2_O (Sigma Aldrich) and followed the same synthesis conditions described for the TAPN catalysts.

### Activity measurements

CO oxidation experiments were carried out using a Varian CP-4900 Micro-GC. 20 mg of sample was packed in between quartz wool inside a stainless-steel reactor tube (I.D. 1/8”). The CO oxidation flow rates consisted of 1.0 ml/min O_2_, 1.5 ml/min CO, and 75 ml/min He (232,500 ml/g_cat_-h). The samples were heated to 300 °C at 2 °C/min and cooled back down to room temperature in the reaction mixture to test for reproducibility. The TOF was calculated using the number of CO_2_ molecules formed per second (rate) divided by the number of active sites (Eq. 1). The active sites were calculated to be the total number of Pt atoms deposited on the ceria surface. 20 mg of a 1wt.%Pt/CeO_2_ used for a CO oxidation run would give ~6.2 × 10^17^ Pt atoms. Activation energies were calculated using steady temperatures while keeping the conversion below 10%.

1 $${\rm{TOF}} = \frac{{{\rm{moles}}\,{\rm{of}}\, {\rm{CO}}_2\,{\rm{produced}}\,{\rm{per}}\,{\rm{second}}}}{{{\rm{moles}}\,{\rm{of}}\,{\rm{Pt}}}}.$$

### HAADF-STEM

Images of the microstructures were acquired using an Aberration Corrected FEI Titan 80–300 Transmission Electron Microscope with an HAADF detector. STEM samples were prepared by dispersing the crushed Pt/CeO_2_ powder on a copper grid with holey carbon film. The powder was transferred onto the Cu grids in a dry form, without the use of a dispersing solution. Additional measurements were done using a JEOL 2010F TEM/STEM operated at 200 kV.

### DRIFTS

The CO oxidation reaction was performed and monitored using DRIFTS and Mass Spectrometry (MS). The infrared spectrometer used was a Tensor 27 from Bruker, coupled with a Praying Mantis™ Diffuse Reflection Accessory from Harrick. The MS used was a ThermoStar GSD 320 T Quadropole Mass Spectrometer (QMS) from Pfeiffer Vacuum, using a Secondary Electron Multiplier (SEM). CO_2_, CO and O_2_ were monitored on the mass-to-charge ratios 44, 28 and 32, respectively. The experiment starts by loading the catalyst in the cell. The temperature is increased to 300 °C under He. Once at 300 °C, a pretreatment with 10%O_2_ was performed for 30 min. The gas was switched to He and backgrounds of the catalysts were taken at 275 °C, 125 °C and 50 °C. CO oxidation was performed for 30 min using a 15 ml/min of 10%CO, 10 ml/min of 10%O_2_ and 15 ml/min of He. CO desorption with He was performed for 10 min using 40 ml/min of He. O_2_ flow after desorption with He was performed for 10 min using 40 ml/min of 10%O_2_. During the reduction step at 275 °C, 40 ml/min of 10%CO, followed by desorption with 40 ml/min of He at the same temperature. Considering the sensitivity of the Pt/CeO_2_ catalysts to CO, when collecting the spectra for the as-synthesized catalysts, oxygen was allowed to flow first for 5 min before introducing CO. Moreover, for the as-synthesized catalysts, switching from CO oxidation to CO desorption with He was performed by stopping first the flow of CO and waiting for 10 s before stopping the flow of O_2_. Considering that CO reacts very easily with O_2_ when the catalysts are activated, for the activated catalysts, switching from CO oxidation to CO desorption with He was performed by stopping first the flow of O_2_ and waiting for 5 s before stopping the flow of CO.

### CO–TPR

CO–TPR measurements were performed using an Autochem 2920 from Micromeritics. Approximately 50 mg of catalyst were used for each experiment. The exhaust line was connected to a QMS from Pfeiffer Vacuum (described above) to analyze the products. CO_2_ formation was monitored on the mass-to-charge ratio 44. The as-synthesized catalysts were exposed to an oxidative pretreatment (300 °C, 10%O_2_, 30 min) before the CO-TPR experiments. The activated catalysts were exposed to an oxidative pretreatment (200 °C, 10%O_2_, 30 min) after activation to replenish the oxygen on the support. The temperature in this case was only 200 °C to avoid possible redispersion of the Pt nanoparticles. A total flowrate of 50 ml/min with a concentration 5%CO, 2.5%Ar was used during the temperature ramp (10 °C/min) and during the data processing the CO_2_ signal was normalized by the signal for the Ar tracer (mass-to-charge ratio 40).

### Raman Spectroscopy

In situ Raman spectra were collected using a Horiba LabRAM HR Raman/FTIR microscope equipped with a 532 nm (Ventus LP 532) laser source and Synapse CCD (Charge Coupled Device) detector, and an in-situ cell (Linkam CCR1000). The presented spectra correspond to the average between three spectra, each one with a 60 s acquisition time. No changes over time were detected in the sample due to laser exposure.

### Operando NAP-XPS

The surface chemistry of Pt/CeO catalysts was characterized in a lab-based SPECS near-ambient pressure X-ray photoelectron spectroscopy (NAP-XPS) system. Operando XPS measurements at pressures of up to 2 mbar are possible due to the incorporation of a reaction cell in the UHV chamber of the NAP-XPS spectrometer. The gases flow through the cell and exit through the outlet port and aperture of the nozzle that interfaces the gas phase in the reaction cell and the vacuum environment of the pre-lens system. The aperture of the 0.3-mm nozzle is close to the spot size of the X-ray beam. A high vacuum in the lens system and electron analyzer (SPECS Phoibos 150 NAP) is maintained via a system of differentially pumped stages. Gases are fed into the NAP-cell via calibrated mass-flow controllers. The overall pressure inside the reaction cell was kept at 2 mbar via a back-pressure controller installed at the outlet of the cell. Prior to the reaction, catalysts were pre-treated in 2 mbar of pure CO (2 ml/min) at 275 °C in order to activate the catalyst. A reaction mixture of 1.5 ml/min of CO and 1 ml/min of O was used, keeping the same ratio between the reactants as for the catalytic studies. High-purity gases were used for the in situ XPS measurements. A standard residual gas analyzer (QMS MKS e Vision 2) placed in the second differential pumping stage allowed following the catalytic activity during XPS measurements. Further technical details about the NAP-XPS system can be found in the literature^60^. Spectra were obtained using monochromatic Al Kα irradiation (1486.6 eV) of an Al anode operating at 50  W. The total acquisition time of the survey spectrum, O 1s, C 1s, Ce 3d, and Pt 4f regions took around 60–70 min. A pass energy of 40 eV with a step size of 0.1 eV and a dwell time of 0.5 s were typically used for acquiring the core-line spectra. Pt 4f and Ce 3d regions were energy-corrected according to the U”’ component of the Ce 3d core line with a characteristic binding energy of 916.7 eV^54,61,62^. The peak position of this component is independent of the Ce to Ce ratio (as long as Ce is present), which makes it possible to calibrate the spectra throughout the experiment. Atomic ratios were estimated following the standard procedure, involving a Shirley background subtraction and raw area normalization by using relative sensitivity factors. Spectral lines were fitted using a symmetric pseudo-Voigt function denoted by a GL (30) line shape in the CasaXPS software. Pt 4f spectra containing a metallic Pt component were fitted using an asymmetric pseudo-Voigt function (LF (0.56, 1.5, 55, 150)). The Ce 3d line was fitted according to a model described elsewhere^61,63^. All the spectra are presented without normalization of the areas. In order to follow possible sintering/segregation of Pt, the Pt/Ce atomic ratios at every temperature were estimated.

### Quasi in-situ XPS (TAPN catalysts)

Quasi in-situ XPS measurements were carried out with a Kratos AXIS Ultra spectrometer, equipped with a monochromatic Al Kα x-ray source and a delay-line detector. Spectra were obtained using the aluminum anode Al *K*α = 1486.6 eV operating at 150 W. The background pressure was in the range of 10^−9^ mbar. Ex situ treatments were performed in the preparation chamber of the spectrometer in 55 mbar of CO at 450 °C for 8 h. After the treatment, the sample was transferred without the exposure to the air to the analysis chamber of the spectrometer, where the spectra were acquired. The acquisition parameters of the spectra were kept as in case of NAP-XPS studies.

### Quasi in-situ XPS (CPA AT catalyst)

A Physical Electronics Quantera Scanning X-ray Microprobe was used to perform the XPS measurements. Monochromatic Al Kα X-ray (1486.7 eV) was used as source for excitation and a spherical section was used as the analyzer. The equipment has a detection system with a 32-element multichannel. The sample was probed by directing the X-ray beam perpendicular to the sample and the detector was at 45°. The spectra were collected using a pass-energy of 69.0 eV. The step size used was 0.125 eV. The Ag 3*d*_5/2_ line, showed a FWHM of 1.0 eV ± 0.05 eV using these conditions. The Cu 2*p*_3/2_ at 932.62 ± 0.05 eV and the Au 4f_7/2_ at 83.96 ± 0.05 eV features were used to calibrate the binding energy scale. Charging was observed during the experiment which was minimized by using low energy electrons at ~1 eV, 20μA and low energy Ar^+^ ions.

### XRD

Experiments were done in a Rigaku SmartLab using a Bragg-Brentano geometry and a DTex high-speed detector that allows for higher signal-to-noise ratio. The diffractograms were collected using the Theta-2Theta scan axis between 25° and 80°, using a rate of 1°/min at a step of 0.01°. The radiation used was Cu *K*-α with a wavelength of 0.154 nm.

### BET surface area

Brunauer, Emmett and Teller (BET) surface area measurements were performed on a Micromeritics Gemini 2360 multipoint analyzer using N_2_ adsorption at −196 ˚C.

## Supplementary information

Supplementary Info

## Data Availability

The authors declare that all the data supporting the findings of this study are available within the paper and its supplementary information file, or from the corresponding author(s) upon request.
