# Supplementary material for: Tuning Pt-CeO2 interactions by high-temperature vapor-phase synthesis for improved reducibility of lattice oxygen
Source: Nat Commun. 2019 Mar 25;10:1358. doi: 10.1038/s41467-019-09308-5 (PMC6433950; doi:10.1038/s41467-019-09308-5)
Supplement: Supplementary file 1 — Supplementary Info [file 41467_2019_9308_MOESM1_ESM.pdf]

Supplementary Information

Tuning Pt-CeO_2 interactions by high-temperature vapor-phase synthesis for improved reducibility of lattice oxygen

Pereira-Hernández et al.

Supplementary Information

Tuning Pt-CeO_2 interactions by high-temperature vapor-phase synthesis for improved reducibility of lattice oxygen

Xavier Isidro Pereira-Hernández[1+], Andrew DeLaRiva[2+], Valery Muravev[3+], Deepak Kunwar[2], Haifeng Xiong[2], Berlin Sudduth[1], Mark Engelhard[4], Libor Kovarik[4], Emiel J.M. Hensen[3,*], Yong Wang[1,5,*] and Abhaya K. Datye[2,*]

[1]Voiland School of Chemical Engineering and Bioengineering, Washington State University, Pullman, WA 99164, USA.

[2]Department of Chemical and Biological Engineering and Center for Micro-Engineered Materials, University of New Mexico, Albuquerque, New Mexico 87131, USA.

[3]Laboratory of Inorganic Materials & Catalysis, Schuit Institute of Catalysis, Eindhoven University of Technology, P.O. Box 513, 5600 MB Eindhoven, The Netherlands.

[4]Environmental Molecular Sciences Laboratory, Pacific Northwest National Laboratory, Richland, WA 99354, USA.

[5]Institute for Integrated Catalysis, Pacific Northwest National Laboratory, Richland, WA 99354, USA.

*Corresponding authors: Prof. A. K. Datye (datye@unm.edu), Prof. Y. Wang (yong.wang@pnnl.gov) and Prof. E.J.M. Hensen (e.j.m.hensen@tue.nl)

[+]These authors contributed equally to this work

Supplementary Figures

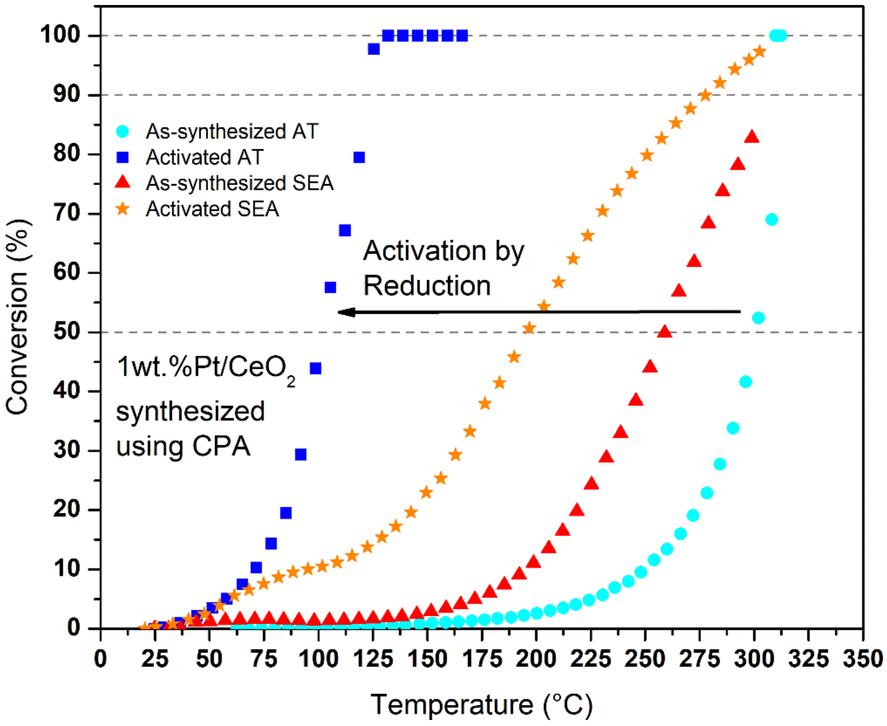

Supplementary Figure 1. CO oxidation light-off curves for 1wt.%Pt/CeO_2 catalysts synthesized by AT and SEA, before and after activation, using CPA as precursor.

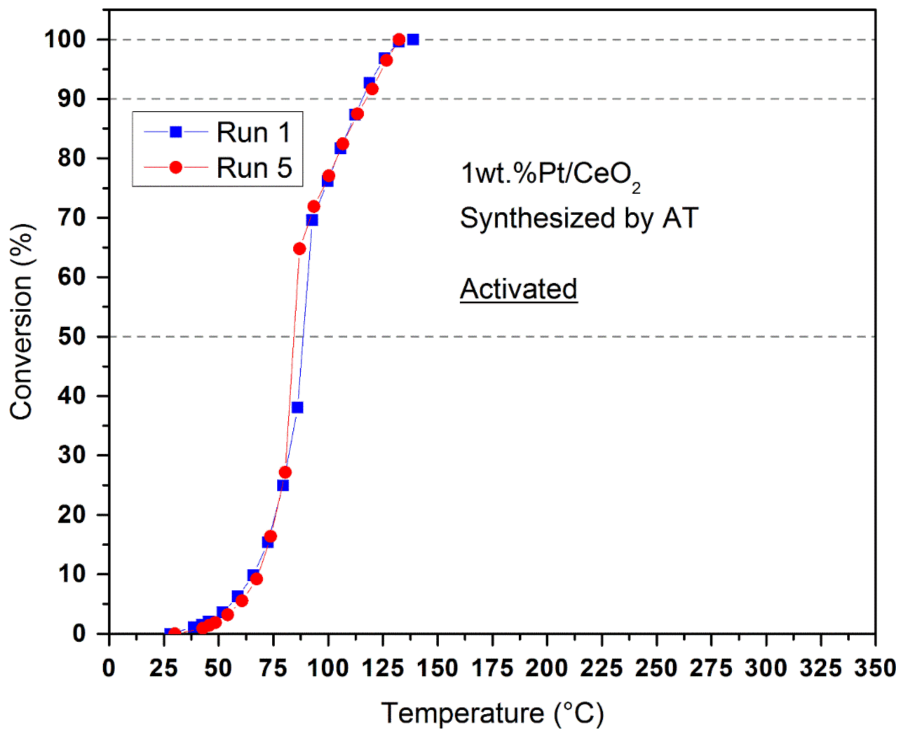

Supplementary Figure 2. Consecutive CO oxidation light-off curves for the 1wt.%Pt/CeO_2 activated AT catalyst, using CPA as precursor.

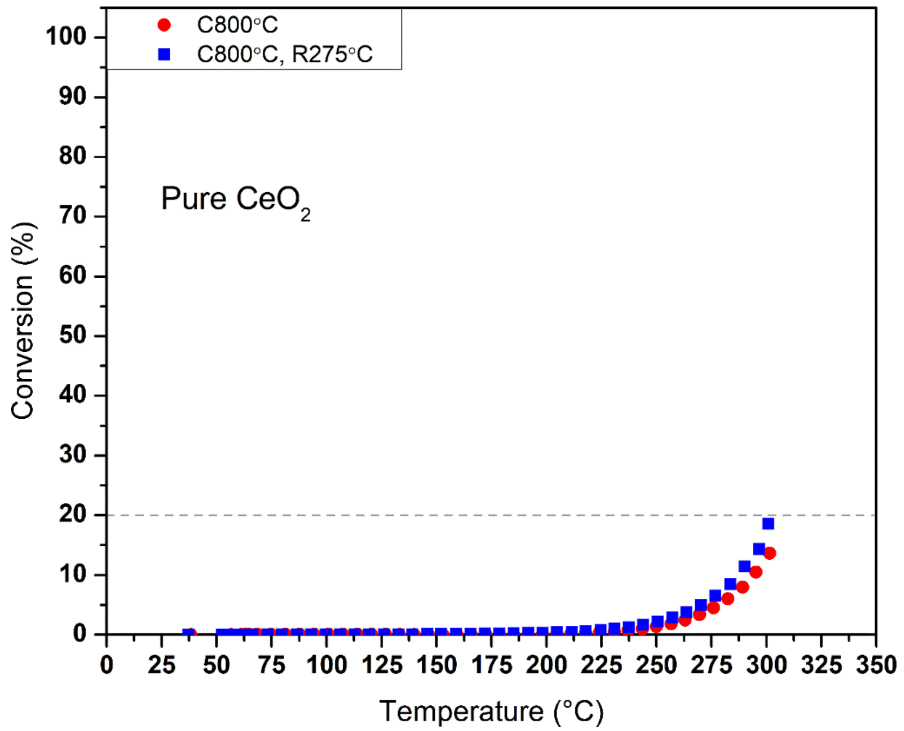

Supplementary Figure 3. CO oxidation reactivity for pure ceria heated to 800 °C (C800°C) and after activation in CO at 275 °C (C800°C, R275°C).

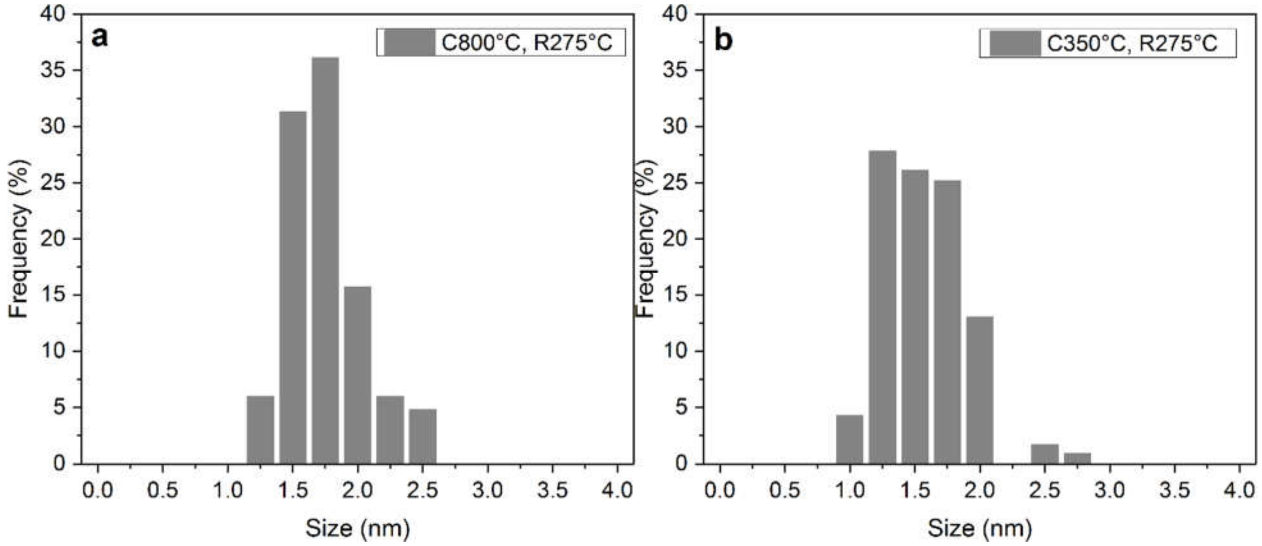

Supplementary Figure 4. Pt Particle size distributions (PSD) for the activated 1wt.% Pt/CeO_2 TAPN catalysts: (a) AT and (b) SEA. The mean particle size and standard deviation for the AT and SEA catalysts are 1.68 ± 0.3 nm and 1.58 ± 0.33 nm, respectively.

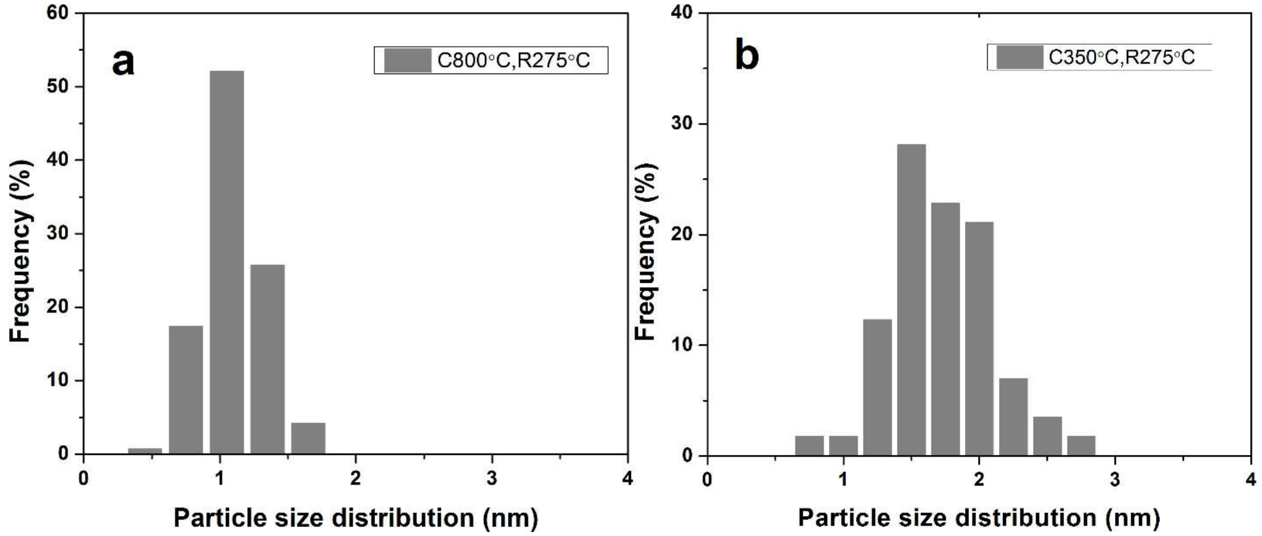

Supplementary Figure 5. Pt Particle size distributions (PSD) for the activated 1wt.% Pt/CeO_2 CPA catalyst: (a) AT and (b) SEA. The mean particle size and standard deviation for the AT and SEA catalysts are 1.05 ± 0.2 nm and 1.72 ± 0.36 nm, respectively.

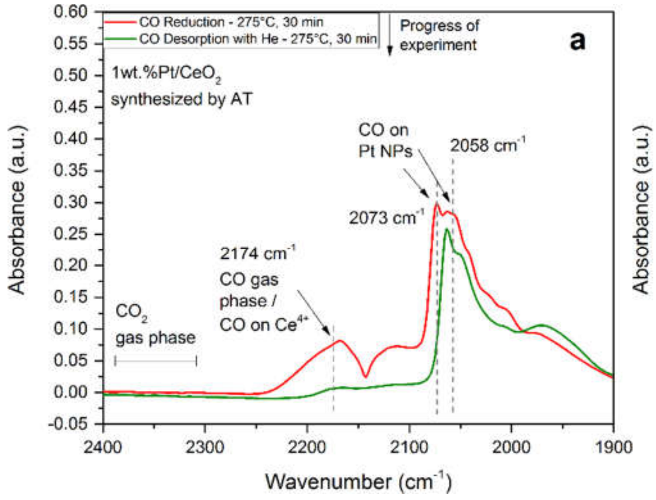

Supplementary Figure 6. CO reduction at 275°C monitored by DRIFTS on the 1wt.% Pt/CeO_2 TAPN catalysts: a) AT, b) SEA. The CO is strongly bound and only partially removed in flowing He.

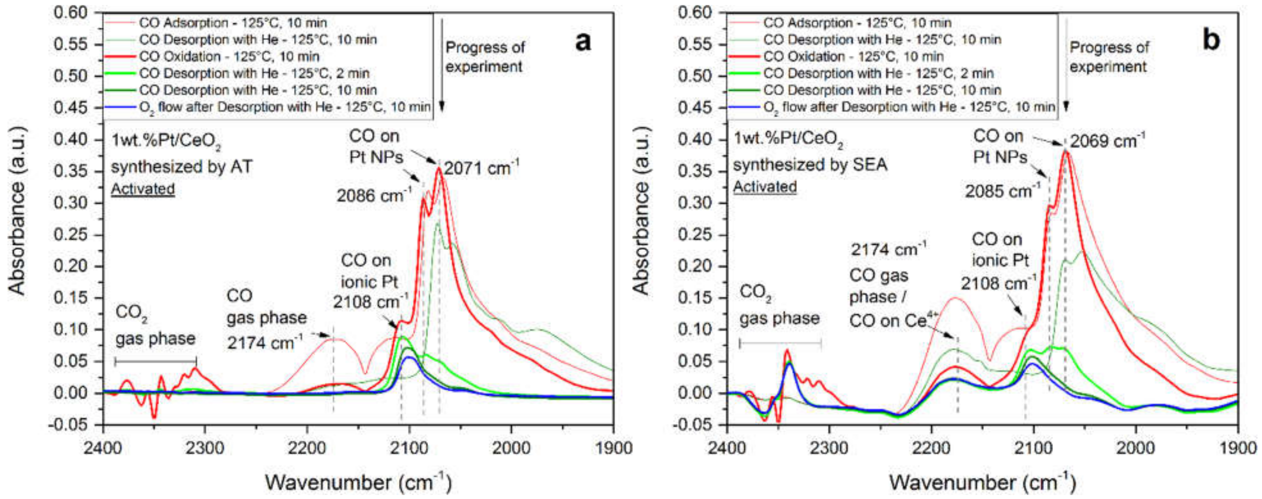

Supplementary Figure 7. CO adsorption/desorption previous to CO oxidation reaction at 125°C monitored by DRIFTS on the activated 1wt.% Pt/CeO_2 TAPN catalysts: a) AT, b) SEA. When CO flow is stopped after CO exposure at 125 °C, the CO band persists, but after CO oxidation the CO is readily removed, presumably because the ceria support has acquired the oxygen that it provides to react with the adsorbed CO.

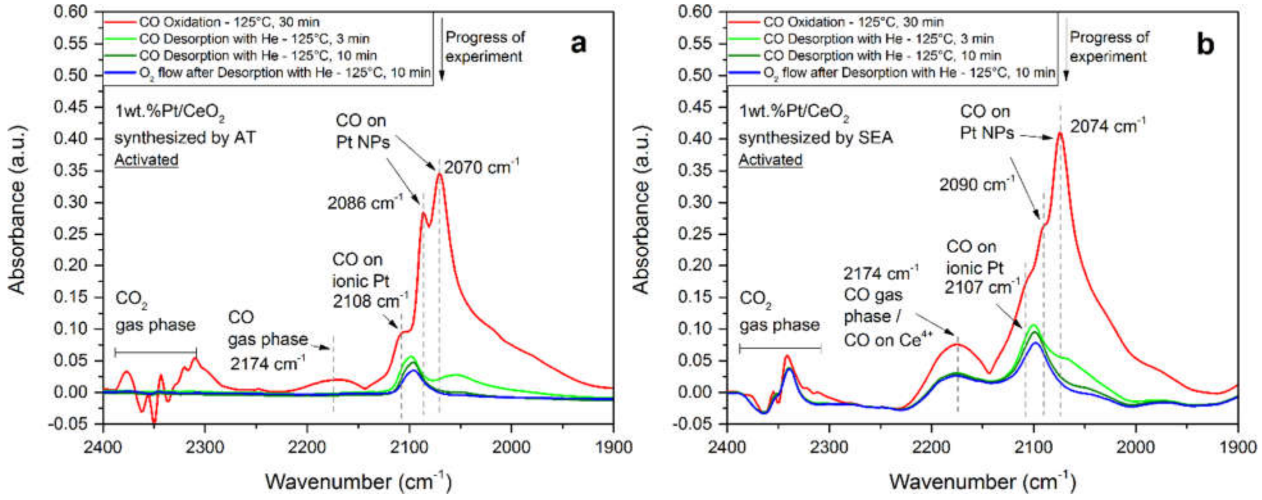

Supplementary Figure 8. CO oxidation reaction at 125°C monitored by DRIFTS on the activated 1wt.% Pt/CeO_2 TAPN catalysts: a) AT, b) SEA. The experiment is similar to that reported in Fig. S7 but involving longer reaction times.

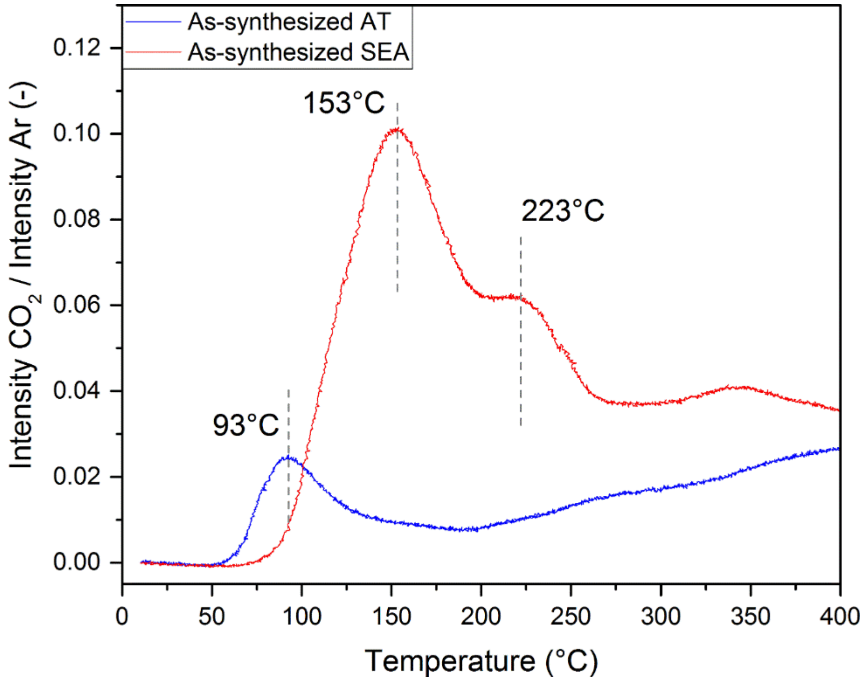

Supplementary Figure 9. CO-TPR of the 1wt.% Pt/CeO_2 TAPN as-synthesized AT and SEA catalysts. The SEA catalyst has a higher surface area since it has not been exposed to 800 °C, explaining in part, the difference in peak areas. The lower temperature peak indicates easier reduction of the trapped ionic Pt and the ceria lattice in the AT catalyst.

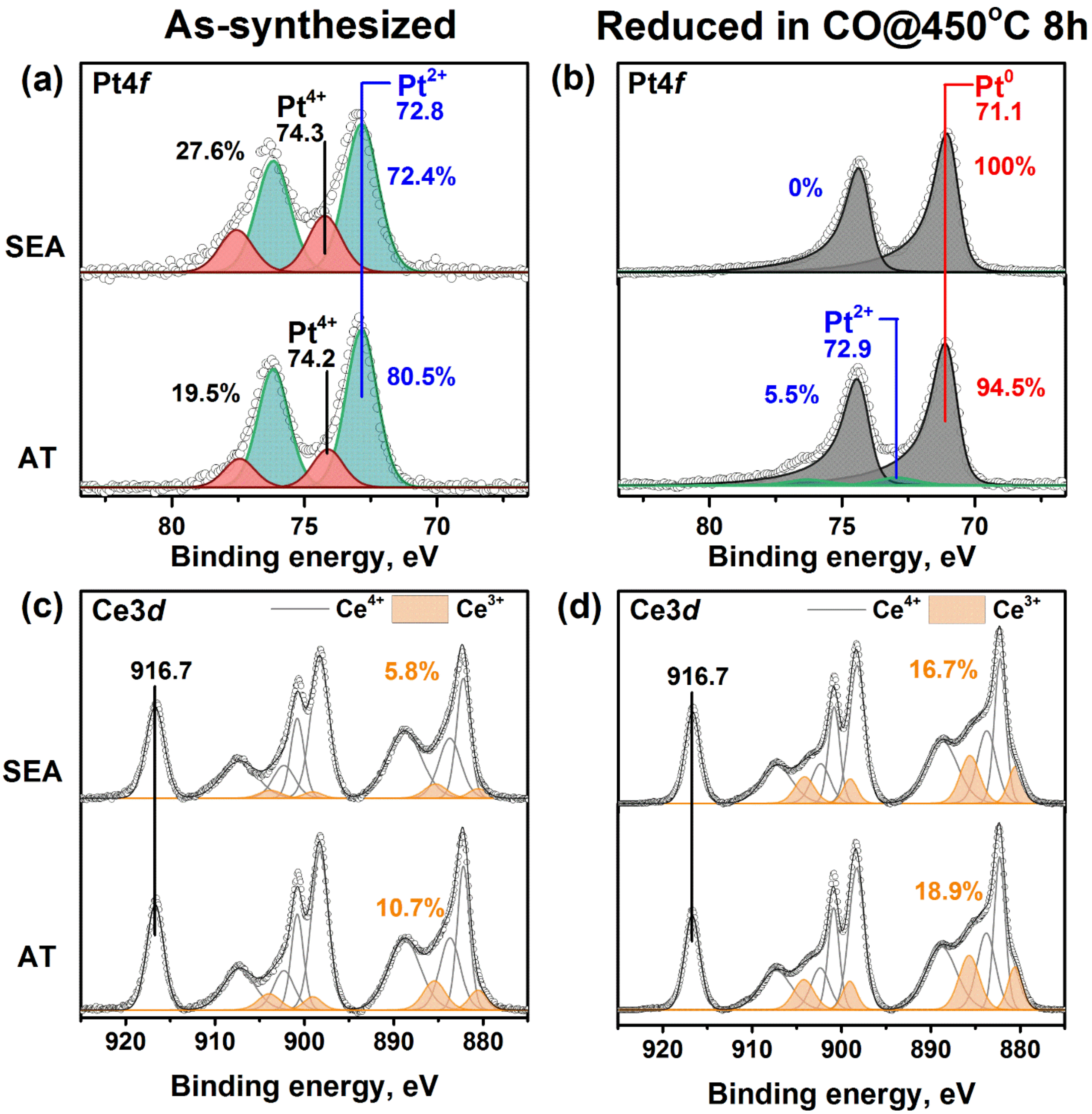

Supplementary Figure 10. XPS results after UHV transfer for 1wt.%Pt/CeO_2 TAPN catalysts: a) Pt4*f* region for the as-synthesized AT and SEA catalysts, b) Ce3*d* region for the as-synthesized AT and SEA catalysts, c) Pt4*f* region for the AT and SEA catalysts after reduction at 450°C for 8 h, d) Ce3*d* region for the AT and SEA catalysts after reduction at 450°C for 8 h. The Pt/Ce ratio changed from 0.025 to 0.021 and 0.017 to 0.038 for the AT and SEA catalysts, respectively, after reduction at 450°C for 8h. The doubling in the Pt/Ce ratio for the SEA catalyst is attributed to the sintering of CeO_2 particles. This is not observed in the AT catalyst since it has already been exposed previously to a temperature of 800°C during synthesis.

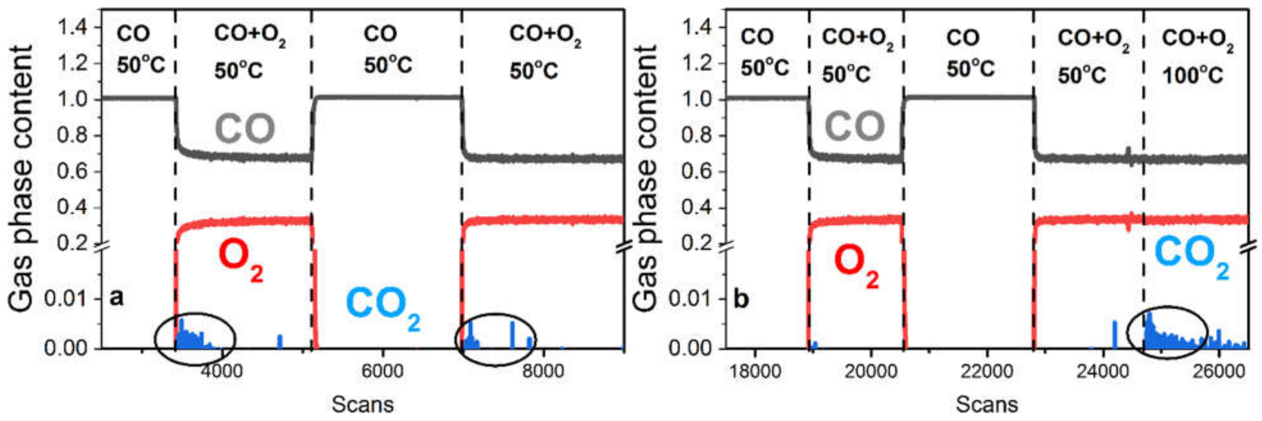

Supplementary Figure 11. Mass spectrometry results during NAP-XPS experiments for the activated 1wt.%Pt/CeO_2 catalysts a) AT, b) SEA, showing the higher reactivity for the AT catalyst at 50 °C. The SEA catalyst shows evidence of reaction only at 100 °C.

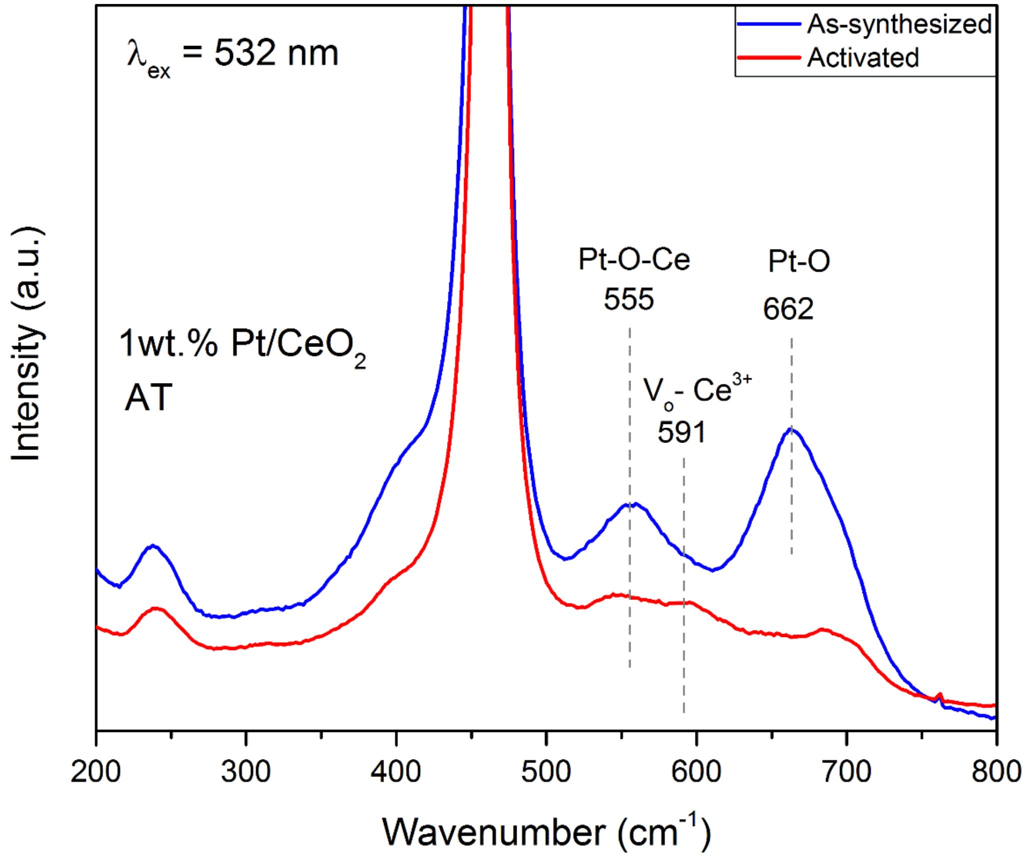

Supplementary Figure 12. Raman spectra of the 1wt.%Pt/CeO_2 TAPN catalyst synthesized by AT, as-synthesized and activated.

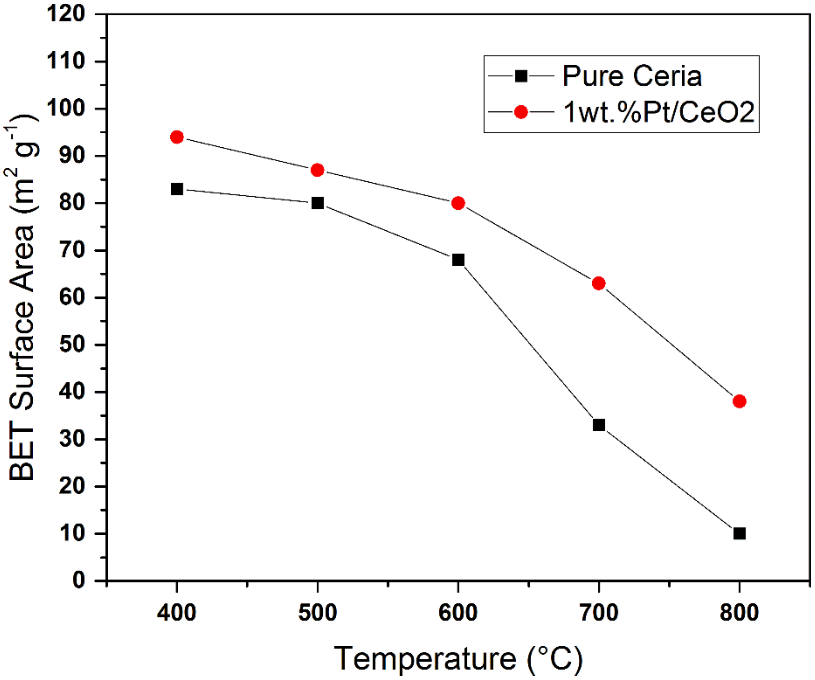

Supplementary Figure 13. BET surface area of pure CeO_2 and the 1wt.% Pt/CeO_2 TAPN catalyst as a function of synthesis temperature.

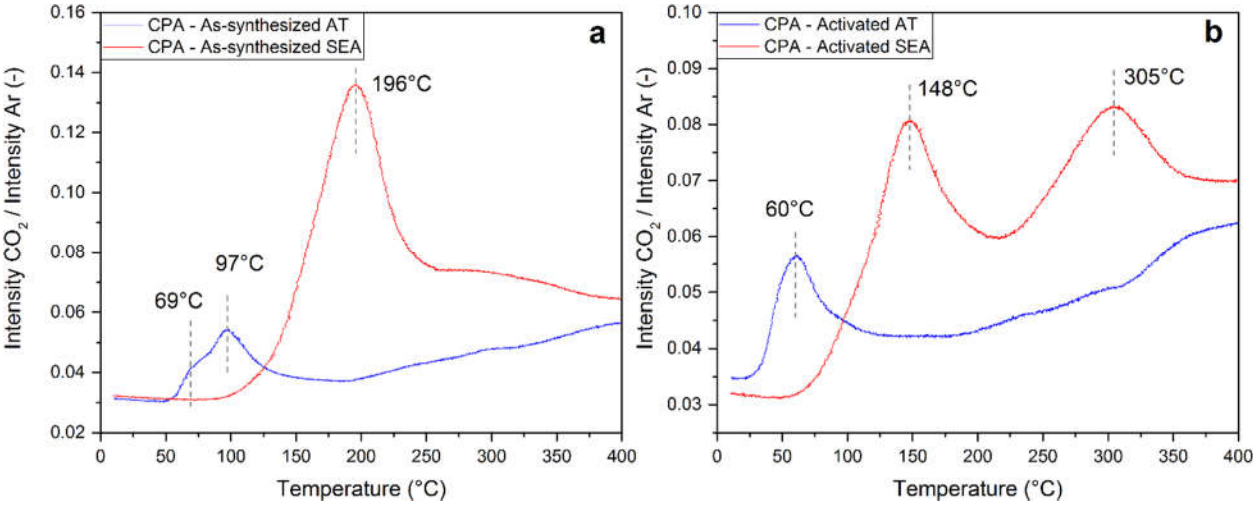

Supplementary Figure 14. CO-TPR of the 1wt.% Pt/CeO_2 CPA catalysts a) as-synthesized AT and SEA, b) activated AT and SEA.

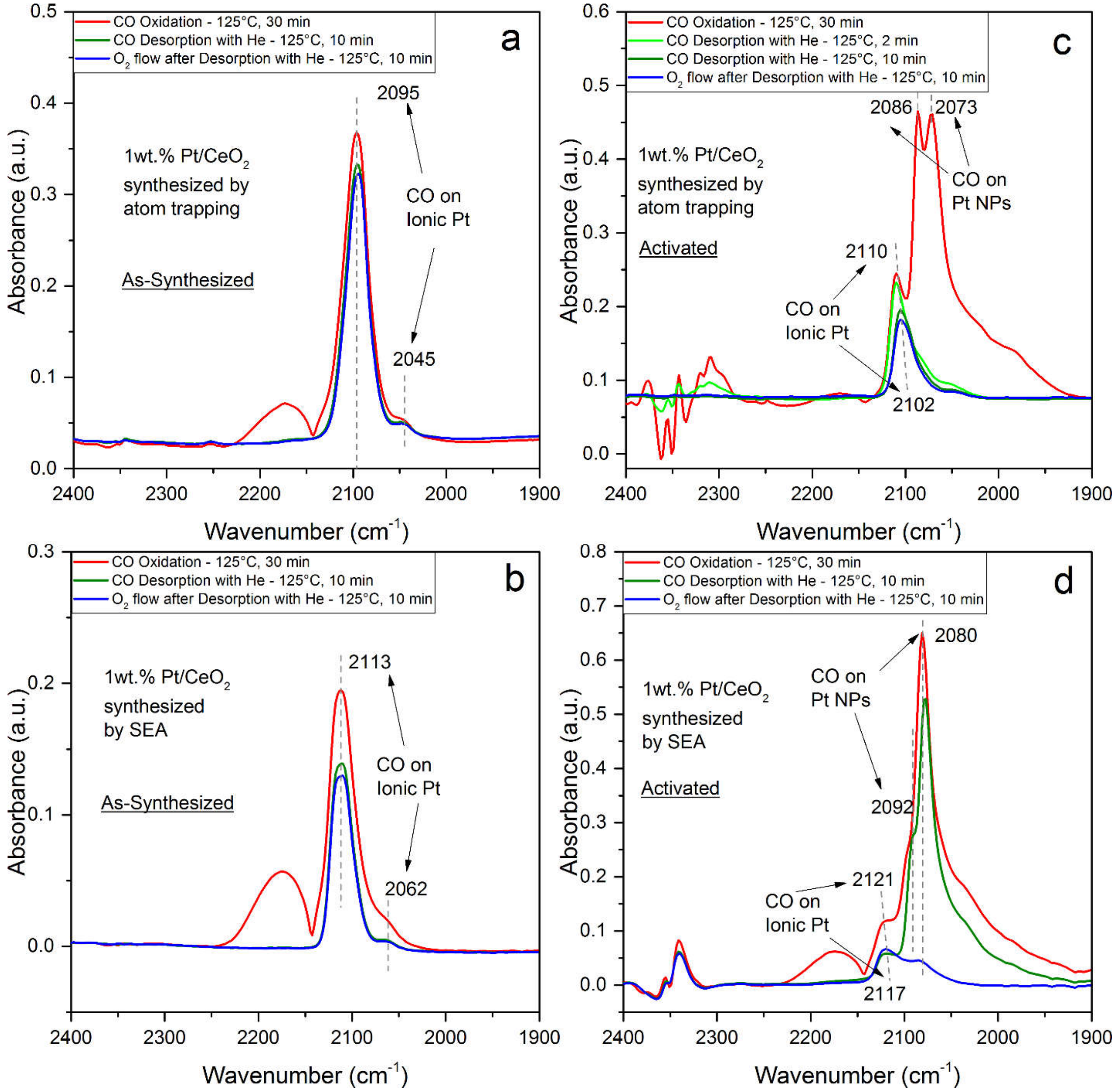

Supplementary Figure 15. CO oxidation reaction at 125°C monitored by DRIFTS on 1wt.% Pt/CeO_2 CPA catalysts: a) as-synthesized AT, b) as-synthesized SEA, c) activated AT, d) activated SEA. The persistence of the CO band on the SEA catalyst after stopping CO is evidence for the lower reactivity of the SEA catalyst prepared via the CPA precursor, already shown in Figure S1.

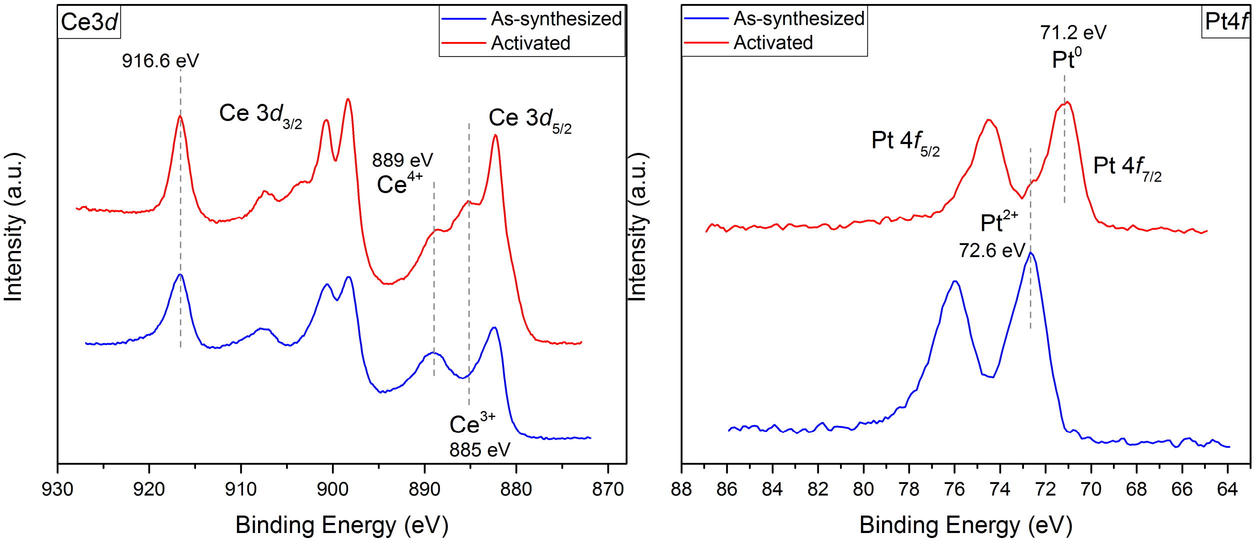

Supplementary Figure 16. XPS spectra of the Ce3*d* and Pt4*f* regions of the 1wt.%Pt/CeO_2 CPA catalyst synthesized by AT, as-synthesized and activated.

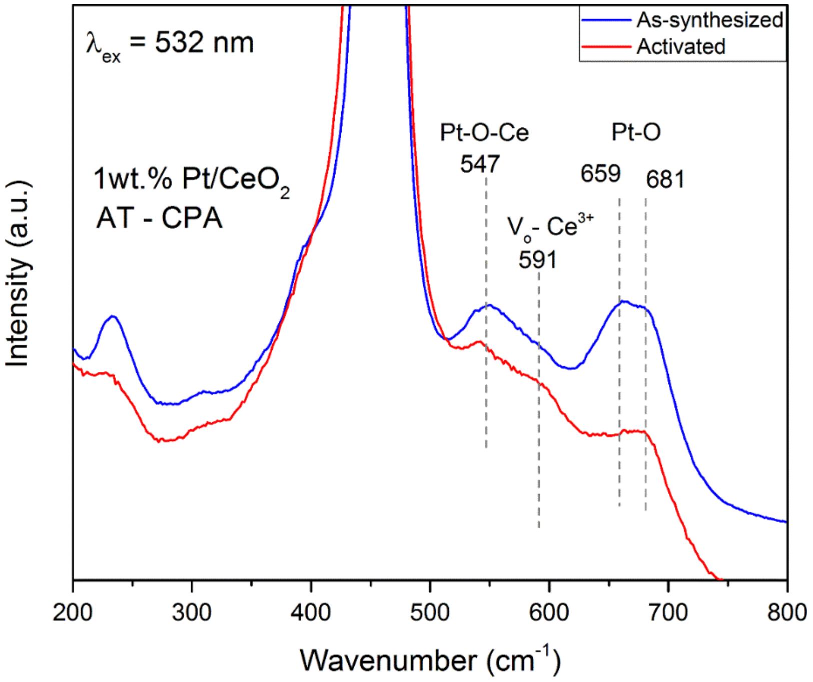

Supplementary Figure 17. Raman spectra of the 1wt.%Pt/CeO_2 CPA catalyst synthesized by AT, as-synthesized and activated.

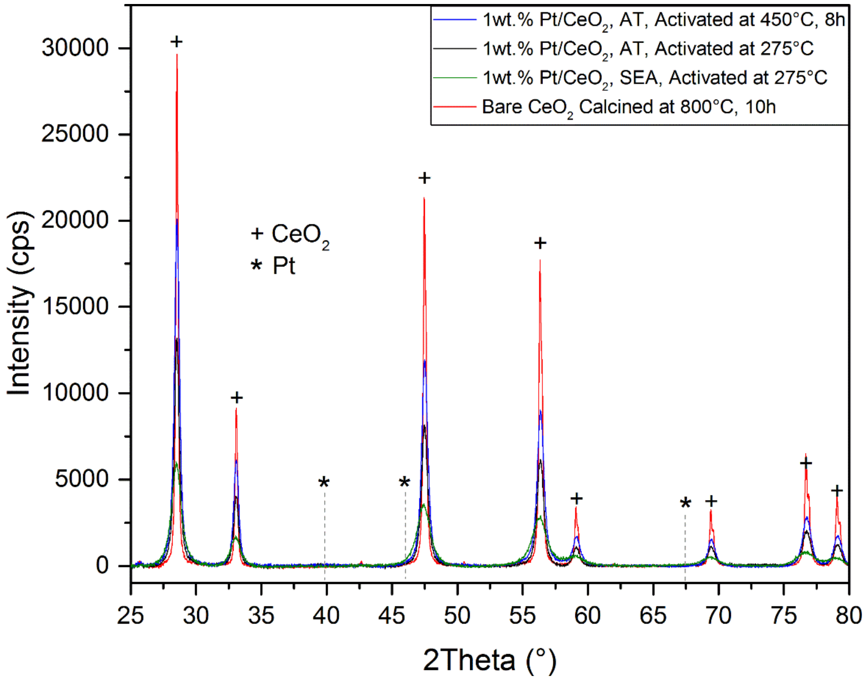

Supplementary Figure 18. XRD pattern for bare CeO_2 calcined at 800 °C for 10 h, the AT and SEA catalysts activated at 275 °C and the AT catalyst activated at 450 °C for 8 h. No diffraction peaks are observed for Pt even after a harsh reduction treatment at 450 °C for 8 h.

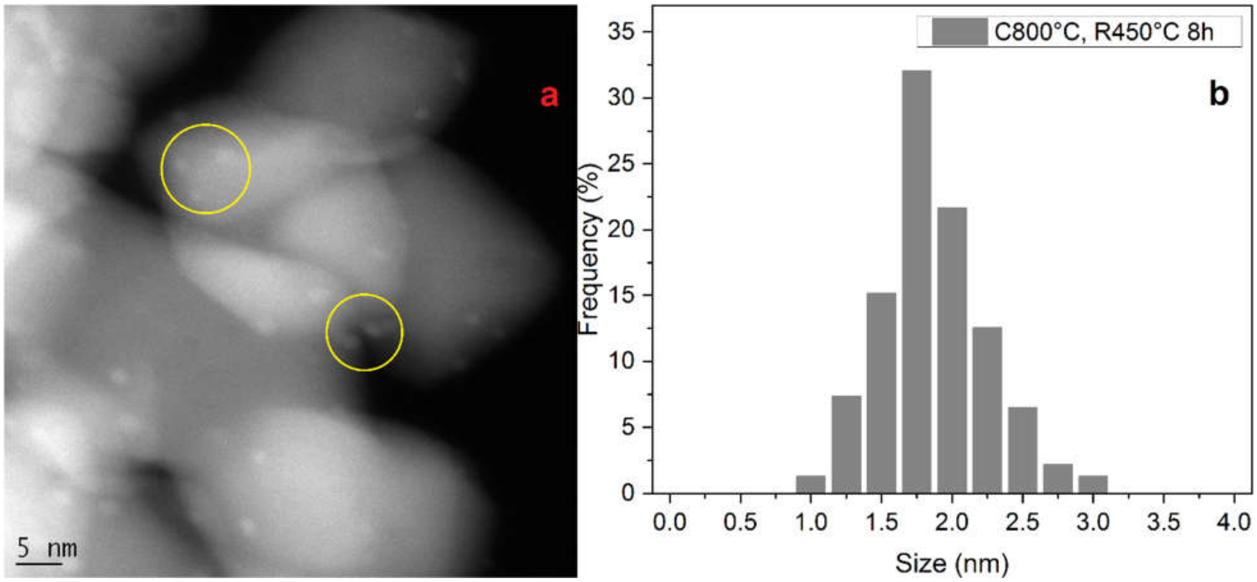

Supplementary Figure 19. Pt particle size after activation of the 1wt.%Pt/CeO_2 TAPN catalyst synthesized by AT at 450 °C for 8 h: a) HAADF-STEM image, b) Pt PSD. The mean particle size and standard deviation are 1.87 ± 0.38 nm.

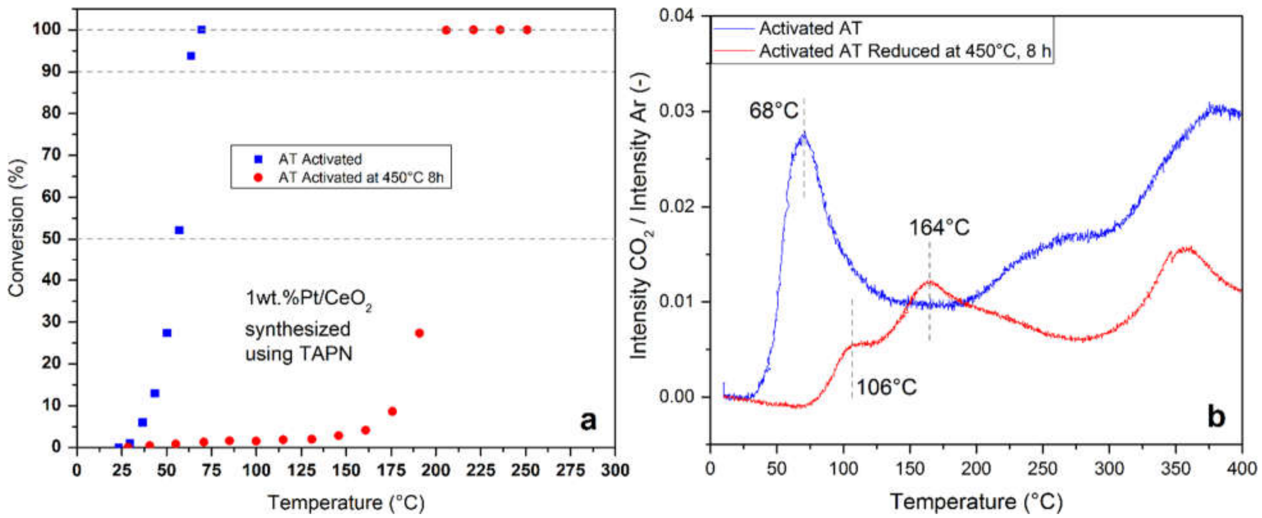

Supplementary Figure 20. Effect of activation at 450 °C on the activity and reducibility of the 1wt.%Pt/CeO_2 catalyst synthesized by AT: a) Light-off curves after activation at 275°C (regular activation treatment) and 450°C for 8 h (harsher activation treatment) and b) CO-TPR after activation at 275 °C (regular activation treatment) and 450 °C for 8 h (harsher activation treatment). The lower reactivity on the 450 °C activated catalyst is correlated with the loss of the low temperature CO-TPR peak.

Supplementary Tables

Supplementary Table 1. Turnover frequencies (TOF) and activation energies (E_A) during CO oxidation for 1wt.%Pt/CeO_2 TAPN catalysts synthesized by AT and SEA.

Catalyst	TOF at 80 °C, s^{-1}	E_A, kJ/mol	T_{50}, °C	T_{90}, °C
As-synthesized AT	0.005	53.5	266	280
Activated AT	0.101	30.1	56	64
As-synthesized SEA	0.013	48.2	250	273
Activated SEA	0.070	34.3	93	120

Supplementary Table 2. Percentages of the intensity of the peak at 2078 cm^{-1} and 2080 cm^{-1} for the activated AT and SEA catalysts in figure 3c and 3d, respectively, during desorption with He, showing the facile removal of the adsorbed CO band on the AT catalyst, explaining how the CO poisoning at low temperatures is mitigated by the active ceria support.

Spectrum	AT	SEA
CO Oxidation - 50°C, 30 min	100	100
CO Desorption with He - 50°C, 3 min	85.3	92.9
CO Desorption with He - 50°C, 5 min	43.2	72.4
CO Desorption with He - 50°C, 8 min	13.1	37.5
CO Desorption with He - 50°C, 10 min	9.5	28.0

Supplementary Table 3. BET surface area of pure CeO_2 and the 1wt.% Pt/CeO_2 TAPN catalyst synthesized by AT as a function of synthesis temperature. Pure CeO_2 retains only 12% of the surface area after being calcined at 800°C while in the 1wt.%Pt/CeO_2 catalyst, CeO_2 retains 40% of the surface area.

Synthesis temperature	400°C	500°C	600°C	700°C	800°C
Surface area CeO_2 ($m^2\ g^{-1}$)	83	80	68	33	10
Surface area 1wt.%Pt/CeO_2 ($m^2\ g^{-1}$)	94	87	80	63	38
